# Effects of Notation Type and Score Difficulty on Eye Movements During Erhu Sight-Reading

**DOI:** 10.3390/jemr19030058

**Published:** 2026-05-27

**Authors:** Siyu Li, Yi Weng, Xiyu Wu

**Affiliations:** 1 Department of Chinese Language and Literature, Peking University, Beijing 100871, China; lllhyun@stu.pku.edu.cn; 2 Laboratory of Language Sciences, Peking University, Beijing 100871, China; 3 Department of Language Science and Technology, The Hong Kong Polytechnic University, Hong Kong 999077, China; yiweng@polyu.edu.hk

**Keywords:** sight-reading, erhu, eye movements, notation type, task difficulty

## Abstract

A parallel notational system comprising number notation and staff notation has long been employed in the pedagogy of traditional Chinese instrumental music. However, it remains unclear how these two representationally distinct notational systems shape performers’ visual-processing strategies. Therefore, using eye-tracking methods, the present study examined the interactive effects of notation type and score difficulty on visual–cognitive processing in a sight-reading task performed on the erhu, a Chinese two-stringed bowed musical instrument. The results showed that increased difficulty generally led to greater fixation-related load and a contracted eye–hand span (EHS). Interaction analyses further indicated that numbered notation was associated with a more favorable eye-movement profile at intermediate difficulty, whereas this pattern was attenuated under difficult score conditions and accompanied by increased processing demands. In contrast, the eye-movement measures in staff notation appeared to be less sensitive to difficulty-related changes than those observed in numbered notation, a pattern that may reflect differences in the representational structures of the two notational systems. Collectively, these findings suggest that notational symbols may modulate sight-reading strategies among trained erhu students, providing preliminary evidence for music reading across different notational systems.

## 1. Introduction

Musical notation serves as a central medium for conveying and organizing musical information. By providing a standardized framework for pitch, rhythm, harmony, and expressive instructions, it forms the foundation of learning and performing music. In musical performance, sight-reading refers to the process of instantly decoding a score and executing a performance with little to no prior preparation [1]—a process that requires a high level of integration across visual–cognitive processing and motor execution. During sight-reading, eye-movement patterns can directly reveal how performers process information and implement performance strategies, thus making eye tracking a key method for investigating music cognition [2,3].

However, the existing research on music sight-reading using eye-tracking methods has largely been conducted within Western instrumental traditions in which staff notation is the dominant representational system, with participants typically drawn from piano, violin, woodwind, and percussion performance [4,5,6,7,8,9]. In contrast, music education in China has long employed two notational systems in parallel—staff notation and numbered notation [10,11]. Compared with staff notation, numbered notation provides a more transparent pitch–symbol mapping, which has facilitated its broad adoption in Chinese music pedagogy, particularly for the transcription and instruction of predominantly monophonic Chinese traditional instruments.

The erhu is a representative bowed string instrument in China that carries substantial historical and cultural significance. Originating from the Tang dynasty *xiqin* (an ancient bowed string instrument), the erhu has undergone centuries of evolution, transforming from a folk instrument used in street performances into a core bowed string instrument in contemporary Chinese national orchestras. The erhu has also established a solo tradition that enjoys international recognition [12,13,14,15,16]. Erhu learners typically begin with numbered notation and gradually transition to staff notation during professional training.

The widespread use of numbered notation has deep historical roots that trace to Rousseau’s early concept of numerical notation, which later was systematized in the nineteenth century through the Galin–Paris–Chevé method, before being introduced into China toward the end of that century [17]. Owing to its concise structure, high symbol recognizability, and low learning threshold, musical notation quickly became widely adopted in both school education and folk music, and it has remained one of the most commonly used notational systems. At present, staff notation predominates in professional conservatory training and in Western classical domains such as orchestral and piano instruction. In contrast, numbered notation is still highly prevalent in Chinese traditional instrumental music, vocal training, and popular songs, and many scores are even printed with staff notation and numbered notation side by side. This context has shaped the notational ecology of Chinese traditional music pedagogy.

**Figure 1:**
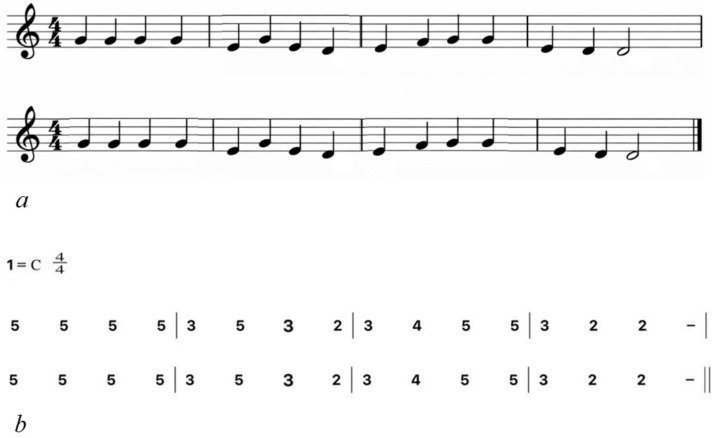
Examples of the two notation types used in the study. (**a**) Staff notation; (**b**) numbered notation. The examples illustrate the different visual-encoding principles of the two systems: spatial pitch representation in staff notation and numerical scale–degree representation in numbered notation.

Staff notation and numbered notation differ markedly in their representations of musical structure (see Figure 1). Staff notation is based on a fixed-pitch framework and uses a two-dimensional spatial layout to depict pitch relationships, harmonic motion, and melodic contour, such that it exhibits higher iconicity and may better support the extraction of musical information from the visual global structure. In contrast, numbered notation relies on a movable-do (tonic-centered) framework that represents scale degrees with numerals. Its symbols are highly distinctive and offer high discriminability, and its decoding tends to be more rules-driven [17,18,19,20,21]. These structural differences imply that learners depend upon different visual cues, spatial referencing strategies, and pitch-processing mechanisms when reading the two notational systems. Numbered notation downplays the spatial mapping between pitch height and visual position, placing lower demands on visuospatial processing while it emphasizes numerical relations and scale–degree decoding. Staff notation, in contrast, requires performers not only to identify symbols but also to process the spatial mapping that links rhythmic structure to action planning. Overall, existing studies have indicated that staff notation and numbered notation differ markedly in their visual structure and information-encoding formats. Such differences are relevant not only to how notation represents musical information but also to how performers interact visually with the score: even changes in the visual spacing of staff notation have been shown to affect sight-reading accuracy [22]. However, it remains unclear how each notation type shapes performers’ cognitive processing during music sight-reading.

Eye tracking can record changes in gaze position over time with millisecond-level temporal resolution, thereby providing a technical basis for capturing the rapidly evolving dynamics of cognitive processing [23]. In music sight-reading, the task inherently requires cross-modal integration of visual symbol recognition, cognitive processing, and motor planning, and accordingly, eye-movement patterns can serve as a process-oriented window into performers’ information processing and strategic choices [2]. Empirical studies have compared experts and non-experts in score-reading tasks and demonstrated that eye-movement measures can differentiate proficiency-related visual strategies [24]. Moreover, changes in notational complexity and visual information density on a musical score have been shown to induce systematic variations in eye-movement parameters such as fixations and regressive saccades, suggesting that these indices are sensitive to task load and the difficulty of symbol processing [25]. Recent studies have further shown that eye-movement measures can be combined with performance-related indices to characterize music-reading expertise and sight-reading behavior more comprehensively [26], while gaze behavior has also been traced across the process from initial sight-reading to performance preparation in string performance contexts [27]. Therefore, eye tracking has been explicitly advocated and increasingly adopted as one of the key methods in music research, enabling observed performance differences to be further interpreted in terms of underlying mechanisms such as attentional allocation, anticipatory processing, and motor control.

In addition, score difficulty has been regarded as a source of task load that shapes sight-reading performance [2]. Score difficulty is influenced not only by the structural complexity of the music, but it is also jointly modulated by the notational format and the motor demands of performance. That said, the existing eye-tracking evidence has focused largely on Western instruments. For example, using a piano sight-reading paradigm, Rosemann et al. [28] showed that increasing difficulty compressed the performer’s eye–hand span (EHS), an effect that became especially pronounced under fast tempi or highly complex performance conditions. In violin sight-reading, Wurtz et al. [7] reported that pieces with higher complexity were associated with lower anticipation in notes, longer fixation duration, and a tendency for more regressive fixations. For woodwind instruments, Zhukov et al. [8] found that as the music score became more difficult, the number of fixations increased, but the duration of each fixation decreased. Similar patterns have also been observed in percussion: Marandola [9] further noted that the number of eye fixations decreased with the augmentation of speed for all percussionists. Beyond instrument-level comparisons, local properties of upcoming notation, such as parafoveal complexity, have also been linked to saccadic and visuo-motor flexibility during sight-reading [29]. Taken together, these studies consistently show that, regardless of instrument type, increased score difficulty tends to compress performers’ look-ahead range and is accompanied by systematic changes in fixation behavior and regressive fixations.

Although previous studies have examined music sight-reading from the perspectives of notation type, score difficulty, cognitive load, and visual strategies, few have investigated the joint effects of notation format (numbered notation vs. staff notation) and difficulty level (intermediate vs. difficult scores) on eye-movement behavior during sight-reading. Consequently, empirical evidence to address three key questions remains lacking. First, do the two notational formats elicit different eye-movement processing patterns under otherwise comparable conditions (a notation effect)? Second, does difficulty level exert a stable influence on performers’ visual-processing strategies (a difficulty effect)? Third, does the impact of notation format vary as a function of difficulty, such that notation-related differences become stronger or weaker at higher levels of difficulty (an interaction effect)?

To explore these questions, the present study took erhu sight-reading as its entry point, and by systematically manipulating notation format and score difficulty while holding other factors constant, we examined differences in performers’ eye-movement behaviors and the underlying cognitive processing mechanisms. In doing so, this work sought to provide more structured and interpretable evidence for understanding the cognitive mechanisms of sight-reading in Chinese traditional instrumental performance.

## 2. Materials and Methods

### 2.1. Participants

A total of 13 undergraduate students majoring in erhu performance were recruited for this study, but ultimately, five participants were excluded due to poor-quality eye-tracking data or inability to complete the experiment. The final sample comprised eight participants, aged 17–23 years (M = 19.5, SD = 2.06), whose erhu training experience ranged from 9 to 13 years (M = 12.13, SD = 1.83). All of the participants had used numbered notation from the beginning of their erhu instruction, yielding 9–13 years of numbered notation experience (M = 12.13, SD = 1.83), whereas their experience with staff notation ranged from 5 to 10 years (M = 7.5, SD = 1.58). Participants were recruited from the China Conservatory of Music and the Central Conservatory of Music and received compensation for their participation. All reported normal or corrected-to-normal vision, and no history of ocular or neurological disorders. The study was conducted in accordance with strict ethical standards, and all participants completed the informed-consent procedure prior to the experiment.

With respect to their music-reading background, the participants had relied primarily on numbered notation before entering conservatory training, using staff notation as a secondary system. After entering the professional music conservatory, they had gradually transitioned to using primarily staff notation, with numbered notation as a supplementary system. In addition, they were receiving systematic solfège and ear-training instruction in staff notation as part of their regular curriculum, further strengthening their proficiency with staff notation.

### 2.2. Equipment and Procedure

The participants’ eye-movement data were recorded using a high-resolution EyeLink 1000 Plus eye tracker (SR Research Ltd., Ottawa, ON, Canada) in remote head-free tracking mode at a sampling rate of 1000 Hz. Because participants needed to play the erhu during the sight-reading task, it was not appropriate to stabilize the head with a chin rest. We therefore used the EyeLink “remote” setup, which does not require head stabilization but tracks a target sticker placed on the participant’s forehead to compensate for head movements during recording. In the present study, the target sticker was placed just above the eyebrow of the tracked eye or on the forehead between the two eyes. The eye tracker was connected to a PC running Experiment Builder (Version 2.4.193, Ottawa, ON, Canada) for real-time data acquisition and storage. Visual stimuli were presented on a Dell P1914S 19-inch monitor (1280 × 1024 pixels; 60 Hz refresh rate; Dell Technologies, Round Rock, TX, USA), and the display settings were configured in accordance with standard eye-tracking experimental protocols.

Before the formal sight-reading task, participants completed a nine-point calibration and validation procedure. Using the default EyeLink settings, the procedure ensured an average validation error of less than 1.0°. During the formal experiment, each of the participants performed on their own erhu while seated in a comfortable posture approximately 60 cm from the computer monitor. Each session consisted of eye-tracker calibration, task execution, and data-quality checks, and lasted approximately 45 min in total. Each participant completed sight-reading of four musical excerpts corresponding to the study’s four experimental conditions: intermediate-difficulty numbered notation, intermediate-difficulty staff notation, difficult-level numbered notation, and difficult-level staff notation. Each excerpt was performed five times, and each session continued until all four excerpts had been completed. To minimize potential data loss and to better capture trial-to-trial fluctuations in performance, data from all five repetitions were retained for subsequent statistical analyses.

### 2.3. Stimuli Materials

**Figure A1:**
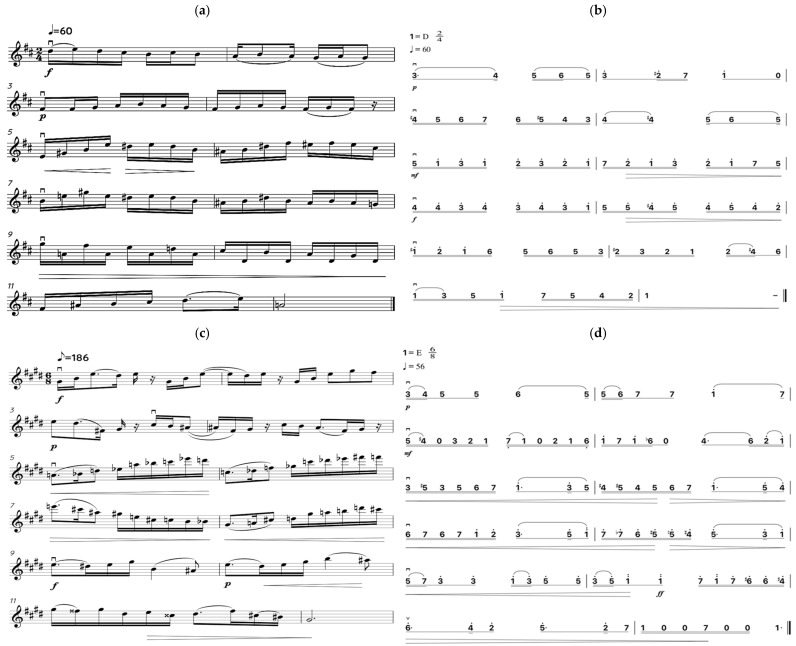
Examples of sight-reading stimuli used in the experiment. (**a**) Intermediate staff notation; (**b**) intermediate numbered notation; (**c**) difficult staff notation; and (**d**) difficult numbered notation. The four panels illustrate the 2 × 2 design crossing notation type and score difficulty.

The experimental stimuli included musical excerpts at two difficulty levels, intermediate and difficult, presented in two notational formats, staff notation and numbered notation. The complete stimulus scores for the four experimental conditions are provided in Appendix A, Figure A1. Score difficulty was determined according to Zhao’s [30] criteria in which mainly consider key signatures, rhythmic variation, and chromatic accidentals. Specifically, the intermediate excerpts generally showed more regular rhythmic organization, a narrower pitch range, a clearer tonal center, and fewer accidentals, whereas the difficult excerpts involved denser note events, more complex rhythmic organization, a wider pitch range, more frequent accidentals, and reduced tonal stability. To provide a clearer description of the testing materials, the main musical and visual-complexity characteristics of the four experimental conditions are summarized in Table 1.

All of the excerpts were entirely unfamiliar to the participants and were selected to represent levels of difficulty that the participants acknowledged as being within their sight-reading capabilities. The staff-notation scores were prepared in Sibelius and exported as PNG images. The numbered-notation versions were created using the iPad application (a digital tool for creating and editing numbered musical notation) [31] and were exported as PNG images. All stimuli were subsequently resized uniformly to 1024 × 768 pixels.

### 2.4. Eye-Movement Analyses

To assess the erhu performers’ sight-reading characteristics under different notational formats and levels of difficulty, this study selected six eye-movement measures: mean fixation duration, fixation count, regressive saccade size, number of regressive fixations, forward saccade size, and eye–hand span. Previous studies have suggested that these eye-movement measures provide informative indices of visual information uptake, processing demands, and anticipatory planning in music sight-reading. Together, these measures capture both the depth and amount of visual information extraction and processing, and they also characterize regressive viewing behavior and saccadic strategies during score reading. During music sight-reading, performers typically use the EHS to look ahead to notes that have not yet been played, thereby gaining time for anticipation and preparation [5,6]. The magnitude of this span is closely related to performers’ skill level, the score’s complexity, and the task’s difficulty [32,33,34,35,36]. In this study, EHS was defined as the note lag between the currently fixated note and the note being executed—that is, the number of notes by which gaze led the hand during performance. This measure provides a direct index of performers’ predictive reading ability for the score in question [7,37,38]. We extracted and calculated these parameters from the collected eye-tracking data, using the eye-movement analysis software DataViewer (Version 3.2.1) [39].

**Figure 2:**
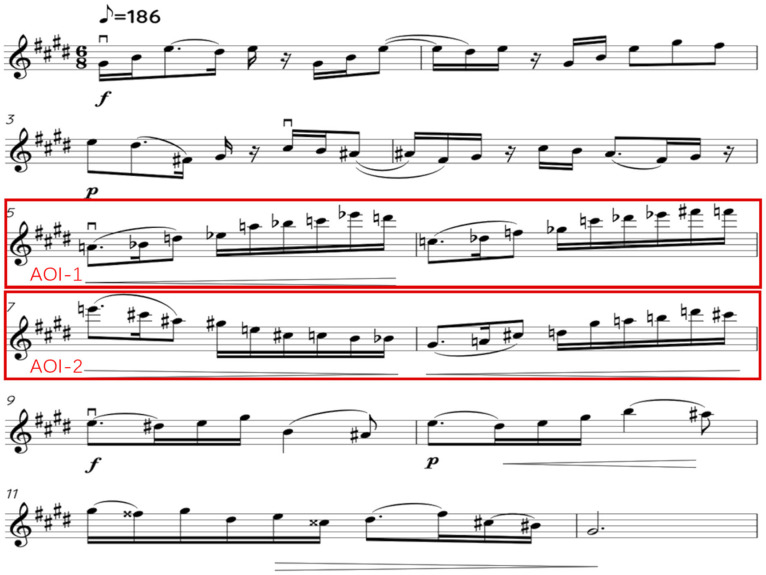
The AOIs used for data analysis during the first sight-reading trial in the difficult staff-notation task were the middle two lines of each score.

In terms of the overall layout of the four excerpts, each score consisted of six lines. Because fixations on the first and last lines are more prone to positional drift, and the first two lines also contain key-signature markings that may induce additional dwell time, we selected only the two middle lines as the areas of interest (AOIs) (see Figure 2). Each line was treated as an independent AOI for calculating the eye-movement measures described above. This AOI definition reduced edge effects and thereby improved the stability and comparability of the subsequent analyses.

In the present study, score difficulty was defined at the level of the complete stimulus excerpt, rather than at the level of individual AOIs. The two AOIs within each excerpt were predefined for eye-movement analysis and were treated as belonging to the same overall difficulty condition. Thus, the analyses focused on how overall score difficulty, together with notation type, influenced the eye-movement measures, rather than on comparisons between AOI1 and AOI2.

Data analyses were conducted in R (version 4.3.2; R Core Team, 2023) [40]. For each of the six AOI-based eye-movement measures, we fitted a linear mixed-effects model with notation type (staff notation vs. numbered notation), score difficulty (intermediate-difficulty vs. difficult-level scores), and their interaction as fixed effects. To account for participants’ prior experience with the two notation systems, training duration in numbered notation and training duration in staff notation were included as covariates. The model can be written as:

$$DV_{\left\{ij\right\}}=\beta_{0}+\beta_{1}Notation_{\left\{ij\right\}}+\beta_{2}Difficulty_{\left\{ij\right\}}+\beta_{3}\left(Notation_{\left\{ij\right\}}\times Difficulty_{\left\{ij\right\}}\right)\;+\beta_{4}Duration{Numbered}_{j}+\beta_{5}Duration{Staff}_{j}+u_{\left\{0j\right\}}+\varepsilon_{\left\{ij\right\}}$$

where $DV_{ij}$ denotes the dependent variable for the i-th observation from the j-th participant; $\beta_{0}$ is the intercept; $\beta_{1}$ and $\beta_{2}$ represent the fixed effects of notation type and score difficulty, respectively; $\beta_{3}$ represents the interaction effect between notation type and score difficulty; $\beta_{4}$ and $\beta_{5}$ represent the effects of training duration in numbered notation and staff notation, respectively; $u_{\left\{0j\right\}}$ is the participant-specific random intercept, and $\varepsilon_{ij}$ is the residual term. The subscript i refers to observations, and j refers to participants.

Model fitting and parameter estimation were performed using the lmer() function in the lme4 [41] package, following a backward model-selection procedure. We first fitted a full model that included all fixed effects and their interaction. Model goodness-of-fit was then compared using likelihood-ratio tests, and the interaction term was retained only if its inclusion significantly improved the model fit. For post hoc analyses, estimated marginal means and pairwise comparisons were conducted with the emmeans package, with Bonferroni correction applied for multiple comparisons. Effect sizes for fixed effects in the linear mixed-effects models were reported as standardized regression coefficients, *β*, obtained by refitting the models with standardized variables. For categorical predictors, *β* values correspond to the displayed model contrasts: staff notation relative to numbered notation for notation type, and difficult-level scores relative to intermediate-difficulty scores for score difficulty. The *β* value for the interaction term represents the standardized contrast for staff notation × difficult-level scores relative to the corresponding reference levels. Effect sizes for post hoc pairwise comparisons were reported as standardized mean differences, *d*, calculated using the residual standard deviation from the corresponding mixed-effects model.

## 3. Results

**Figure 3:**
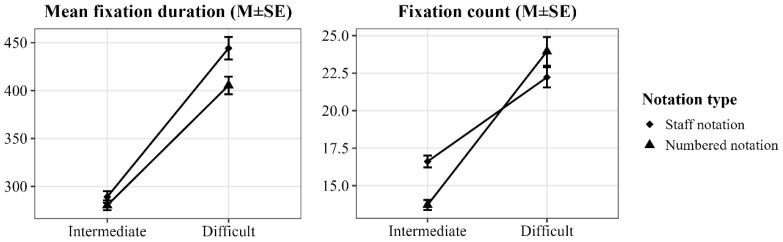

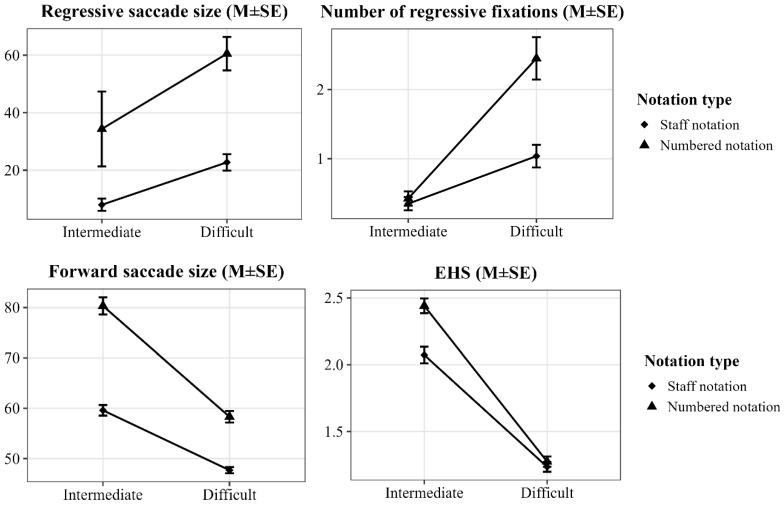
Eye-movement measures across notation and difficulty conditions. Circles indicate staff notation, and triangles indicate numbered notation. The panels show mean fixation duration, fixation count, regressive saccade size, number of regressive fixations, forward saccade size, and eye–hand span. Error bars represent standard errors of the mean. EHS = eye–hand span.

Table 2 summarizes the means and standard deviations of each eye-movement measure across the two notation types and score difficulty levels. Figure 3 plots each eye-movement measure as a function of score complexity, with staff notation shown as circles and numbered notation as triangles.

### 3.1. Mean Fixation Duration

With the mean fixation duration measures, likelihood-ratio tests indicated that adding the main effect of score difficulty (χ^2^ = 138.13, *p* < 0.01, *β* = 1.21, 95% CI [1.00, 1.41]) and the interaction between notation type and score difficulty (χ^2^ = 3.98, *p* = 0.046, *β* = 0.29, 95% CI [0.00, 0.58]) to the model significantly improved model fit. Post hoc pairwise comparisons showed that difficult scores elicited significantly longer mean fixation durations than intermediate-difficulty scores did (*t* = 18.61, *p* < 0.01, *d* = 2.08, 95% CI [1.74, 2.43]). The significant interaction arose because, at the intermediate-difficulty level, mean fixation duration did not differ by notation type (*p* = 0.41), whereas at the difficult level, the staff notation produced significantly longer fixation durations than the numbered notation did (*t* = −3.64, *p* < 0.01, *d* = 0.576, 95% CI [0.203, 0.949]). 

### 3.2. Fixation Count

With the fixation count, likelihood-ratio tests showed that inclusion of the main effects of score difficulty (χ^2^ = 194.49, *p* < 0.01, *β* = 1.45, 95% CI [1.25, 1.66]) and notation type (χ^2^ = 15.61, *p* < 0.01, β = 0.41, 95% CI [0.21, 0.62]), as well as their interaction (χ^2^ = 19.85, *p* < 0.01, *β* = −0.66, 95% CI [−0.95, −0.37]), significantly improved the model fit. Post hoc pairwise comparisons indicated that the significant main effect of difficulty was driven by a reliable increase in the fixation count for difficult scores (*t* = 15.72, *p* < 0.01, *d* = 1.71, 95% CI [1.38, 2.03]). In contrast, post hoc tests for the main effect of notation type were not significant (*t* = 1.13, *p* = 0.26, *d* = −0.13, 95% CI [−0.15, 0.40]). Post hoc analyses of the interaction further revealed that, for both notation types, difficult scores elicited significantly more fixations than intermediate scores did (both *p*s < 0.01). When the score was of intermediate difficulty, staff notation produced significantly more fixations than numbered notation did (*t* = −3.95, *p* < 0.01, *d* = −0.63, 95% CI [−1.00, −0.25]), but when the score was difficult, numbered notation produced significantly more fixations than staff notation did (*t* = 2.35, *p* = 0.02, *d* = 0.37, 95% CI [0.00, 0.74]). 

### 3.3. Regressive Saccade Size

With the regressive saccade size measure, likelihood-ratio tests indicated that inclusion of the main effects of score difficulty (χ^2^ = 6.48, *p* < 0.01, *β* = 0.38, 95% CI [0.08, 0.68]) and notation type (χ^2^ = 6.38, *p* < 0.01, *β* = −0.39, 95% CI [−0.69, 68]) significantly improved the model fit. Post hoc pairwise comparisons showed that difficult scores elicited significantly larger regressive saccade sizes than the intermediate scores did (*t* = 2.79, *p* = 0.01, *d* = 0.31, 95% CI [0.08, 0.54]). In addition, numbered notation produced significantly larger regressive saccade sizes than staff notation did (*t* = 4.38, *p* < 0.01, *d* = 0.49, 95% CI [0.25, 0.72]).

### 3.4. Number of Regressive Fixations

With the number of regressive fixations, likelihood-ratio tests indicated that adding the main effect of score difficulty (χ^2^ = 69.16, *p* < 0.01, *β* = 1.08, 95% CI [0.83, 1.34]), the interaction between notation type and score difficulty (χ^2^ = 15.08, *p* < 0.01, *β* = −0.72, 95% CI [−1.08, −0.35]), and the covariate of training duration of numbered notation (χ^2^ = 6.33, *p* = 0.01, β = −0.27, 95% CI [−0.48, −0.06]) significantly improved the model fit. Post hoc pairwise comparisons showed that the main effect of score difficulty was driven by a significant increase in the number of regressive fixations for difficult scores (*t* = 7.88, *p* < 0.01, *d* = 0.88, 95% CI [0.62, 1.14]). Post hoc analyses of the interaction further revealed that, for both notation types, difficult scores elicited significantly more regressive fixations than intermediate scores did (both *p*s < 0.01). When the score was of intermediate difficulty, the number of regressive fixations did not differ by notation type (*t* = 0.31, *p* = 0.76), whereas when the score was difficult, staff notation produced significantly more regressive fixations than numbered notation did (*t* = 5.80, *p* < 0.01, *d* = 0.92, 95% CI [0.58, 1.23]). Training duration in numbered notation showed a negative association with the number of regressions (*b* = −0.276, SE = 0.110, 95% CI [−0.56, 0.01]).

### 3.5. Forward Saccade Size

With forward saccade size, likelihood-ratio tests indicated that inclusion of the main effects of score difficulty (χ^2^ = 256.63, *p* < 0.01, *β* = −1.39, 95% CI [−1.56, −1.22]) and notation type (χ^2^ = 227.54, *p* < 0.01, *β* = −1.30, 95% CI [−1.47, −1.13]), as well as their interaction (χ^2^ = 27.06, *p* < 0.01, β = 0.64, 95% CI [0.40, 0.88]), significantly improved the model fit. Post hoc pairwise comparisons showed that the significant main effect of score difficulty was driven by larger forward saccade sizes for intermediate-difficulty scores than for difficult scores (*t* = 17.45, *p* < 0.01, *d* = 1.95, 95% CI [1.61, 2.3]), whereas the significant main effect of notation type was driven by larger forward saccade sizes in connection with the numbered notation than with the staff notation (*t* = 16.13, *p* < 0.01, *d* = 1.80, 95% CI [1.47, 2.14]). Post hoc analyses of the interaction of the score difficulty and the notation type further indicated that, for both numbered notation and staff notation, intermediate-difficulty scores elicited significantly larger forward saccade sizes than difficult scores did (both *p*s < 0.01). Likewise, at both intermediate-difficulty and difficult-difficulty levels, numbered notation produced significantly larger forward saccade sizes than staff notation did (both *p*s < 0.01).

### 3.6. Eye–Hand Span (EHS)

With the eye–hand span measurements, likelihood-ratio tests indicated that inclusion of the main effects of both the score difficulty (χ^2^ = 23.78, *p* < 0.01, *β* = −1.72, 95% CI [−1.88, −1.55]) and notation type (χ^2^ = 48.55, *p* < 0.01, *β* = −0.54, 95% CI [−0.71, −0.37]), as well as their interaction (χ^2^ = 48.15, *p* < 0.01, β = 0.48, 95% CI [0.25, 0.72]), significantly improved the model fit. Post hoc pairwise comparisons showed that intermediate-difficulty scores elicited a significantly larger EHS than difficult scores did (*t* = 24.47, *p* < 0.01, *d* = 2.74, 95% CI [2.35, 3.12]), and that the numbered system of notation produced a significantly larger EHS than staff notation did (*t* = 4.97, *p* < 0.01, *d* = 0.55, 95% CI [0.27, 0.84]). Post hoc analyses of the interaction further indicated that, for both notation types, intermediate scores yielded a significantly larger EHS than difficult scores did (both *p*s < 0.01). Furthermore, when the score was of intermediate difficulty, numbered notation produced a significantly larger EHS than staff notation did (*t* = 6.35, *p* < 0.01, *d* = 1.00, 95% CI [0.62, 1.39]), but when the score was difficult, the participants’ EHS measures did not differ by notation type (*p* = 0.50). 

## 4. Discussion

Employing an erhu sight-reading task, the present study demonstrated that notation type, score difficulty, and their interactions all significantly modulated musical performers’ eye-movement patterns. Overall, the numbered notation system tended to elicit lower fixation counts, larger forward saccade sizes, a larger EHS, and larger regressive saccade sizes, whereas the staff notation system was associated with higher fixation counts, smaller forward saccade sizes, and a more locally incremental and stable progression through the scores. Meanwhile, increasing the level of difficulty generally led to longer mean fixation durations and higher fixation counts, along with a larger number of regressive fixations, and it was accompanied by smaller forward saccade sizes and a reduced EHS. In the following sections, we discuss the effects of notation type, the effects of score difficulty, and the mechanisms underlying their interactions, and then we distill the consequent theoretical and pedagogical implications.

### 4.1. The Effects of Notation Type on Sight-Reading Behavior

The present study’s results indicate that music notation type exerts an important influence on eye-movement behavior during erhu sight-reading. Specifically, statistically significant main effects of notation type were observed in the models for fixation count, regressive saccade size, forward saccade size, and EHS. Compared with the staff notation type, when performers were sight-reading a numbered notation, they displayed a lower fixation count—thus suggesting that they were able to extract and integrate note information more efficiently within a given amount of viewing time [34,42,43]. At the same time, the numbered notation type was associated with larger forward saccade sizes, a larger EHS, and larger regressive saccade sizes, thus indicating that sight readers under the numbered-notation condition tended to scan the score in a more anticipatory manner while also engaging in longer-range look-backs for local correction [6,37,42].

We interpret these findings as suggesting that the two notational systems elicit different processing strategies during sight-reading. Numbered notation is visually centered on numerals (scale degrees), whose symbols are often more discriminable. Across comparative studies of notational systems, higher discriminability has been associated with faster visual identification and discrimination performance [17,18,19,20,21]. Accordingly, the lower fixation count observed under numbered notation—together with larger forward saccade size and a larger EHS—may indicate that the study’s erhu performers were more inclined to advance through the score using wider-span scanning to meet the immediate task demands. At the same time, the numbered notation type yielded significantly larger regressive saccade size. In reading and music-notation research, regressive eye movements are commonly taken as overt indicators of rechecking, integration, or error correction of previously processed information. Consistent with this interpretation, the covariate analysis further showed that longer total training duration in numbered notation was associated with fewer regressive fixations, suggesting that greater familiarity with numbered notation may reduce the need for repeated checking during sight-reading. In contrast, staff notation is characterized by encoding pitch via two-dimensional spatial position and is therefore often higher on the dimension of iconicity [17,18,19,20,21]. From this perspective, the smaller forward saccade size and smaller regressive saccade size—together with higher fixation count—can be understood as reflecting a more locally incremental mode of processing that relies on continuous decoding and stable spatial anchoring.

### 4.2. The Effects of Score Difficulty on Sight-Reading Behavior

We found that increasing the score difficulty significantly altered the performers’ sight-reading strategies, as reflected across all six eye-movement measures: mean fixation duration, fixation count, number of regressive fixations, regressive saccade size, forward saccade size, and eye–hand spans. For both the staff notation and numbered notation systems, as the scores shifted from simpler to more complex, the performers generally showed longer mean fixation durations and higher fixation counts, thus indicating that both decoding musical symbols and planning actions require more time and cognitive resources under conditions of higher information density and more complex note groupings [8,44]. At the same time, the sight-readers’ forward saccade size and EHS decreased significantly with increasing score difficulty, suggesting that under a heavier task load, the performers were less able to maintain anticipatory scanning over a broader region of the score. Visual processing thus gradually shifted from a more predictive strategy toward a more cautious, localized, segment-by-segment mode of processing [25,33]. In addition, regressive eye movements became more frequent and larger as the score difficulty increased, implying that when they were sight-reading complex passages, the performers were more likely to return to previously read locations for error correction or for rechecking rhythmic and pitch structures [7,45].

Notably, although the erhu is a traditional Chinese bowed string instrument, its sight-reading eye-movement patterns showed a highly consistent direction of change with increasing difficulty, similar to what has been reported for Western instruments such as the piano and violin. This may reflect a general effect of difficulty on sight-reading across instruments: as difficulty increases, EHS contracts and look-ahead scanning weaken; mean fixation duration lengthens, and fixation count increases; and regressive behavior becomes more frequent [2,6,7,28,46]. These findings may therefore be regarded as preliminary evidence that difficulty-related changes in sight-reading share certain common features across different instrumental contexts.

### 4.3. Interaction Between Notation Type and Score Difficulty

The interaction between notation type and score difficulty was also of particular interest in this study. As score difficulty increased from intermediate to complex, the moderating effect of notation type showed different trends across five eye-movement measures: mean fixation duration, fixation count, number of regressive fixations, forward saccade size, and EHS.

First, interaction effects between notation type and difficulty were also evident for two indices of local processing—mean fixation duration and number of regressive fixations. Under the intermediate-difficulty condition, differences between staff notation and numbered notation were small for both measures, and the overall levels were comparable, suggesting that when information density is still manageable, performers can complete sight-reading rapidly and stably. As score difficulty increased to the complex level, however, both the mean fixation duration and the number of regressive fixations rose markedly, and those increases were more pronounced under the numbered notation. For complex scores, numbered notation yielded longer mean fixation durations than the staff notation type did, along with a higher number of regressive fixations, indicating that under the numbered notation system, performers needed to devote more time to item-by-item decoding of pitch and rhythm and to rely on frequent look-backs to verify note localization and fingering mappings [7,25,46]. From a cognitive-processing perspective, the staff notation system may still support a certain degree of pattern-based and chunked processing in complex passages by providing a stable spatial framework, thereby reducing the need for repeated checking of individual symbols. In contrast, complex numbered-notation scores often contain numerous accidentals, ornaments, and non-canonical rhythmic patterns, which can disrupt the perceptual fluency of the melodic structure. As a result, performers may need to switch repeatedly among numerals, pitch interpretation, and fingering configurations, and they may be able to maintain accurate decoding only by prolonging fixations and increasing regressive eye movements. This leads to a heavier local processing load under high-difficulty conditions.

Second, forward saccade size and regressive saccade size showed broadly similar directional changes with increasing score difficulty, yet the statistical results indicated that their interaction patterns were not the same. Forward saccade size was significantly larger with staff notation than with numbered notation at the intermediate-difficulty level, whereas when difficulty increased to the complex level, forward saccade size decreased significantly in both types of notations and converged to similar values, thereby yielding a significant interaction between notation type and score difficulty. This pattern suggests that as informational load increases, the forward-scanning advantage observed for staff notation is compressed to a greater extent: performers in the staff-notation condition are forced to shift from wider-span look-ahead scanning to a more local browsing strategy. In contrast, numbered notation processing already relies on relatively local saccade control at the intermediate level, so the additional contraction induced by increased difficulty is comparatively limited [28,37]. From a cognitive-processing perspective, staff notation provides a stable spatial reference for pitch, which at intermediate difficulty facilitates rapid extraction of phrase contour via larger saccades. Numbered notation, however, requires symbolic conversion among numerals, pitch interpretation, and fingering configurations, and thus naturally depends more on short-span, point-by-point decoding. Consequently, under complex scores, both systems are ultimately driven toward a “narrow look-ahead” mode dominated by local processing [10,11,20]. Notably, the pattern for regressive saccade size differed from that for forward saccade size. For both the staff notation and numbered notation systems, regressive saccade size increased substantially as score difficulty rose from intermediate to complex, indicating that higher difficulty triggers broader look-backs and more frequent rechecking in both notational systems [45]. However, the two curves were largely parallel, implying similar difficulty-related increases across notations; accordingly, the interaction was not statistically significant. In other words, when performers needed to return to previously read regions to correct or confirm information, notation-specific differences were overshadowed by the shared high demands for processing. Under such conditions, regressive behavior appears to reflect a general response to overall difficulty rather than a differentiated adaptation to notational format.

Turning to fixation count, an opposite-direction interaction between notation type and score difficulty emerged. Under the intermediate condition, fixation count was slightly higher for staff notation than for numbered notation, suggesting that when task demands are still manageable, performers adopt a relatively more stable retrieval strategy in staff notation and require more fixations to map pitch information onto fingering configurations. In contrast, because numbered notation encodes relative pitch with numerals, performers can often scan phrases with fewer fixations when the mode is familiar, and the passage structure is relatively regular [18,19,21]. However, as difficulty increased to the complex level, fixation count rose significantly for both notational formats [8,25,28], but the increase was more pronounced for the numbered notation, causing the two curves to cross. Under complex scores, the numbered notation yielded a higher fixation count than staff notation did, indicating that under a high load, the visual-processing demands imposed by the two systems were effectively reversed. From a cognitive-processing perspective, under intermediate difficulty, numbered notation provides a relatively compact representation of relative pitch, with visually distinctive symbols. Performers can therefore recognize melodic patterns quickly and may rely on templatelike extraction for common modes and frequently used pitch patterns, which allows them to browse local phrases with fewer fixations. Under complex difficulty, however, numbered-notation scores not only increase substantially in note density but may also involve less-familiar modes and numerous chromatic accidentals. Scale–degree relationships and functional anchoring become less transparent, forcing performers to advance through the score with denser, more short-lived fixations, thereby markedly increasing their overall fixation count. In contrast, staff notation may still allow performers to capture a wider range of structural information within a single fixation in high-difficulty passages, thus reducing the number of fixations needed to complete the same segment. Accordingly, the interaction in fixation count suggests that as difficulty increases, staff notation becomes relatively more conducive to global pattern recognition and structural integration, whereas numbered notation is more likely to push sight-reading toward a mode characterized by high-frequency, local decoding.

A clear interaction between notation type and score difficulty also emerged for eye–hand span. Under the intermediate-difficulty condition, EHS was significantly larger in staff notation than in numbered notation, indicating that at comparable complexities, staff notation supports a wider look-ahead window: performers can plan upcoming movements on the basis of notes farther ahead when reading staff notation. When difficulty increased to the difficult level, however, EHS decreased markedly for both notations and converged to a similar level, leaving the original notation-type difference no longer significant. This pattern suggests that, under high task-load conditions, performers’ look-ahead behavior may become more constrained, reducing the extent to which notation-specific advantages can be expressed [28,37,38].

At the intermediate-difficulty level, staff notation directly depicts melodic contour through the spatial positions of pitches on the staff, thus facilitating the integration of several consecutive notes within a single look-ahead episode. Numbered notation, in contrast, requires additional conversions among numerals, scale–degree interpretation, and fingering mapping, thus likely constraining processing to shorter segments and to proceeding in a more item-by-item manner. As a result, fewer notes can be previewed in advance [18,19,20,21]. When the score becomes more complex, both systems combine high note density with intricate rhythmic structures, placing substantial demands on performers’ working memory and attentional resources. Performers, therefore, tend to narrow their look-ahead range and shift toward note-by-note processing that focuses on the current and immediately upcoming notes, thereby ultimately yielding similarly short-range look-ahead patterns in eye–hand spans.

### 4.4. Theoretical Implications and Practical Applications

From an eye-tracking perspective, the present study reveals differentiated visual-processing mechanisms in erhu performers when reading staff notation versus when reading numbered notation, thus providing new evidence for cognitive models of music reading across multiple notational systems. Previous research on musical sight-reading has largely been grounded in Western staff-notation traditions, while it often overlooks the long-standing dual-notation learning practice that has coexisted in Chinese music education [10,11]. Our findings suggest that staff notation and numbered notation differ not only in representational format but also in the cognitive strategies they elicit, in a systematic way. Numbered notation tends to promote a more global-scanning mode with anticipatory planning, whereas staff notation relies more on continuous decoding and locally stable spatial anchoring. These differences indicate that music reading is not a single, universal processing routine that remains invariant across notational formats; rather, it is modulated by the formal properties of the notational system itself. Different notational systems can prompt performers to adopt distinct strategies for organizing and extracting information, thereby giving rise to format-specific sight-reading patterns. In this sense, the observed differences may be related to how each notation helps performers coordinate the decoding of currently fixated material with the preparation of upcoming musical actions [47].

At the same time, the present findings may offer useful implications for erhu instruction, particularly for trained students who are learning to coordinate numbered notation and staff notation in sight-reading. Within the present sample and task conditions, the two notational systems were associated with different eye-movement patterns and attentional demands. Therefore, erhu instruction may benefit from treating numbered notation and staff notation as complementary rather than fully interchangeable systems. Numbered notation may be useful for supporting melodic identification in relatively manageable sight-reading contexts, whereas systematic and stepwise practice in staff-notation reading may help students strengthen spatial-position mapping and fingering-position anchoring. In addition, the negative association between total training duration in numbered notation and the number of regressive fixations suggests that extended and systematic exposure to a notational system may ease the demand for repeated rechecking and error correction during sight-reading. These pedagogical implications should, however, be interpreted cautiously and further evaluated in teaching contexts involving learners at different proficiency levels. Second, our study shows that score difficulty has a pronounced impact on anticipatory processing, thus suggesting that instruction may benefit from explicitly cultivating students’ look-ahead skills through eye–hand span-oriented training. For example, practice routines could incorporate strategy drills such as “silently previewing one bar ahead” or “playing while reading the next line,” thereby strengthening predictive execution and improving continuity of the performance.

### 4.5. Limitations and Future Directions

Nevertheless, although the present study revealed key characteristics of visual processing during erhu sight-reading, several limitations should be acknowledged. First, the present study was conducted with a relatively small sample of erhu performers (**N = 8**) under controlled laboratory conditions, which may limit the generalizability of the findings to broader groups of performers and to more natural performance situations. Future studies should therefore include a substantially larger and more diverse group of erhu performers and should further examine whether the present findings can be replicated in more naturalistic sight-reading contexts.

Second, the present study did not include performance-related measures such as error rate, note-level accuracy, or tempo deviation. Future work should combine these measures with eye-movement data to better model the relationship between visual–cognitive processing and motor execution during sight-reading.

Third, the present study focused only on two notational formats—staff notation and numbered notation—whereas Chinese musical traditions also include structurally distinct systems such as *jianzipu* (a tablature-based notation system) and *gongchepu* (a traditional pitch-based notation system). In addition, the musical materials used in the present experiment were controlled excerpts designed for the eye-tracking task. Future work could extend the comparison to a broader range of notational systems and include more complex or naturalistic musical materials, so as to further validate sight-reading strategies across different musical and cultural contexts.

## 5. Conclusions

This study employed an eye-tracking system to examine how music notation type and score difficulty may shape visual processing during erhu sight-reading tasks. The results identified differences between the two notational formats when score difficulty was controlled. Overall, numbered notation was associated with a lower fixation count, larger forward saccade size, larger eye–hand span, and larger regressive saccade size. These patterns may reflect a relatively more anticipatory reading strategy involving broader visual scanning and longer-range error correction. In contrast, staff notation was associated with a higher fixation count, smaller forward saccade size, and smaller regressive saccade size, a pattern that may indicate a reading mode characterized by more continuous decoding and locally stable spatial anchoring. Score difficulty appeared to modulate visual processing across both notational formats. From intermediate- to high-difficulty scores, performers showed longer mean fixation durations and higher fixation counts, together with reduced EHS and forward saccade sizes, as well as increased numbers of regressive fixations and larger regressive saccade sizes. This pattern suggests that, under higher task demands, sight-reading may shift from a relatively anticipatory and globally planned processing mode toward a more cautious, localized monitoring and error-correction approach. The interaction effects further suggest that numbered notation may support more efficient visual processing for some eye-movement measures at the intermediate-difficulty level, whereas this advantage appears to be reduced under high-difficulty conditions. By contrast, eye-movement patterns during staff-notation reading appeared to be less strongly modulated by score difficulty in the present data.

Taken together, these findings suggest that the representational structure of music notation may influence performers’ attentional allocation and anticipatory planning during erhu sight-reading. The present findings provide preliminary evidence for understanding music reading across different notational systems and may offer useful implications for the sequencing of notation type and score difficulty in erhu sight-reading training.

## Figures and Tables

**Table 1:** Musical and visual-complexity characteristics of the sight-reading stimuli.

Condition	Complexity Profile
Intermediate staff notation	Regular duple-meter grouping, moderate pitch range, few accidentals, and relatively low symbol density.
Intermediate numbered notation	Regular duple-meter grouping, clear phrase organization, moderate pitch range, few accidentals, and relatively sparse numerical-symbol density.
Difficult staff notation	Compound-meter grouping, denser beamed-note groups, wider pitch range, and more frequent accidentals.
Difficult numbered notation	Compound-meter grouping, higher numerical-event density, wider pitch range, and more frequent accidentals.

**Table 2:** Eye-tracking measures by notation type and score difficulty. Values are presented as mean ± standard deviation for each experimental condition.

Notation Type	Score Difficulty	Mean Fixation Duration	Fixation Count
Staff notation	Intermediate	289.09 ± 6.03	16.61 ± 0.40
	Difficult	444.22 ± 11.66	22.23 ± 0.67
Numbered notation	Intermediate	280.33 ± 5.21	13.71 ± 0.34
	Difficult	405.44 ± 9.19	23.95 ± 0.96
Notation type	Score difficulty	Regressive saccade size	Number of regressive fixations
Staff notation	Intermediate	7.98 ± 2.13	0.35 ± 0.10
	Difficult	22.74 ± 2.86	1.04 ± 0.16
Numbered notation	Intermediate	34.35 ± 13.0	0.42 ± 0.10
	Difficult	60.51 ± 5.80	2.45 ± 0.31
Notation type	Score difficulty	Forward saccade size	Eye–hand span EHS
Staff notation	Intermediate	59.6 ± 1.04	2.07 ± 0.06
	Difficult	47.69 ± 0.63	1.23 ± 0.04
Numbered notation	Intermediate	80.33 ± 1.70	2.44 ± 0.06
	Difficult	58.31 ± 1.13	1.27 ± 0.04

## Data Availability

The data presented in this study are available on request from the corresponding author, due to privacy restrictions. The data are not publicly available to ensure compliance with participant confidentiality agreements and relevant data protection legislation.
